# Translation of Atherosclerotic Plaque Phase-Contrast CT Imaging from Synchrotron Radiation to a Conventional Lab-Based X-Ray Source

**DOI:** 10.1371/journal.pone.0073513

**Published:** 2013-09-09

**Authors:** Tobias Saam, Julia Herzen, Holger Hetterich, Sandra Fill, Marian Willner, Marco Stockmar, Klaus Achterhold, Irene Zanette, Timm Weitkamp, Ulrich Schüller, Sigrid Auweter, Silvia Adam-Neumair, Konstantin Nikolaou, Maximilian F. Reiser, Franz Pfeiffer, Fabian Bamberg

**Affiliations:** 1 Institute of Clinical Radiology, Ludwig-Maximilians-University Hospital, Munich, Germany; 2 Chair of Biomedical Physics, Technical University of Munich, Munich, Germany; 3 European Synchrotron Radiation Facility (ESRF), Grenoble, France; 4 Synchrotron Soleil, Gif-sur-Yvette, France; 5 Center for Neuropathology, Ludwig-Maximilians-University Hospital, Munich, Germany; 6 Institute of Anatomy, Ludwig-Maximilians-University, Munich, Germany; 7 Institute of Materials Research, Helmholtz-Zentrum Geesthacht, Geesthacht, Germany; University of Notre Dame, United States of America

## Abstract

**Objectives:**

Phase-contrast imaging is a novel X-ray based technique that provides enhanced soft tissue contrast. The aim of this study was to evaluate the feasibility of visualizing human carotid arteries by grating-based phase-contrast tomography (PC-CT) at two different experimental set-ups: (i) applying synchrotron radiation and (ii) using a conventional X-ray tube.

**Materials and Methods:**

Five ex-vivo carotid artery specimens were examined with PC-CT either at the European Synchrotron Radiation Facility using a monochromatic X-ray beam (2 specimens; 23 keV; pixel size 5.4 µm), or at a laboratory set-up on a conventional X-ray tube (3 specimens; 35-40 kVp; 70 mA; pixel size 100 µm). Tomographic images were reconstructed and compared to histopathology. Two independent readers determined vessel dimensions and one reader determined signal-to-noise ratios (SNR) between PC-CT and absorption images.

**Results:**

In total, 51 sections were included in the analysis. Images from both set-ups provided sufficient contrast to differentiate individual vessel layers. All PCI-based measurements strongly predicted but significantly overestimated lumen, intima and vessel wall area for both the synchrotron and the laboratory-based measurements as compared with histology (all p<0.001 with slope >0.53 per mm^2^, 95%-CI: 0.35 to 0.70). Although synchrotron-based images were characterized by higher SNRs than laboratory-based images; both PC-CT set-ups had superior SNRs compared to corresponding conventional absorption-based images (p<0.001). Inter-reader reproducibility was excellent (ICCs >0.98 and >0.84 for synchrotron and for laboratory-based measurements; respectively).

**Conclusion:**

Experimental PC-CT of carotid specimens is feasible with both synchrotron and conventional X-ray sources, producing high-resolution images suitable for vessel characterization and atherosclerosis research.

## Introduction

Advanced imaging techniques have greatly contributed to our understanding of disease processes and play a pivotal role in the diagnostic work-up of major disease states. The performance of X-ray computed tomography (CT) and magnetic resonance imaging (MRI) for vascular imaging has developed rapidly during the past years resulting in robust acquisitions of the vascular tree with high diagnostic accuracy [1]. Despite these advances, one of the inherent drawbacks of CT is its limited soft-tissue resolution, which is crucial for characterization of atherosclerotic plaque composition [2], while MRI has excellent soft tissue contrast but is limited to large and stationary vessels such as the carotid arteries [3,4]. Consequently, a modality that provides enhanced soft tissue contrast at high spatial resolution would greatly advance the field and help to improve patient care.

There are very early results indicating that X-ray phase-contrast imaging (PCI) may provide improved soft-tissue resolution compared to conventional absorption CT by exploiting phase-shift rather than absorption information [5,6]. Any phase-shift occurs when photons pass through different components of material. There are different phase contrast imaging methods that detect the phase-shift indirectly from measured intensities (e.g. propagation-, crystal analyzer-, or grating interferometer-based). It has been shown that these phase-contrast imaging techniques provide improved and complementary images as compared to standard X-ray absorption methods [6–10]. At this early stage, however, most clinically-motivated PCI tomography studies are carried out with monochromatic and brilliant X-ray sources at large-scale synchrotron facilities, thus impeding preclinical evaluation. Recently, by applying a grating-based interferometer, the modality has been successfully transferred from synchrotron sources to conventional laboratory-based broadband X-ray tubes [11–14]. Grating-based phase-contrast tomography (PC-CT) has already been used to image cartilage/ tendons [14], heart tissue [13], ex-vivo brain tissue [9,15] as well as breast cancer [16] with conventional X-ray tubes.

The aim of this proof-of-principle study was to determine the potential of grating-based PC-CT with a conventional X-ray tube for carotid vessel wall imaging in comparison with histopathology and bench-marking results obtained at a synchrotron source. Our hypothesis was that PC-CT would allow differentiation and quantification of vessel layers in both, the laboratory and synchrotron setting, and that PC-CT in both set-ups provides improved soft tissue contrast compared to absorption CT.

## Materials and Methods

### Ethics Statement

This project was designed as a post-mortem, ex-vivo feasibility study. This HIPAA-compliant study was conducted in accordance with the Declaration of Helsinki and was approved by the institutional review board (Ethikkommission of the Ludwig-Maximilian-University, Munich). In all cases patients or the next of kin provided written informed consent to donate tissue for research purposes.

### Study Design and Experimental Overview

Five human adult carotid artery specimens with a length of 2-3 cm were harvested post-mortem and examined either (i) at the European Synchrotron Radiation Facility (ESRF) in Grenoble, France (n=2) or (ii) at a laboratory-based experimental set-up with a conventional X-ray tube (n=3). The two arteries scanned at the ESRF were distal external carotid artery autopsy specimens with very early atherosclerotic changes; the three carotid arteries scanned with the conventional X-ray tube were carotid artery autopsy specimens with advanced atherosclerotic disease which included the carotid bifurcation and portions of the common, internal and external carotid arteries. Subsequently, all specimens were histologically processed. PC-CT images were matched with the corresponding histological sections every 2.0 mm and analysis of different vessel wall parameters was performed.

### Grating-based Phase-Contrast CT

**Figure 1:**
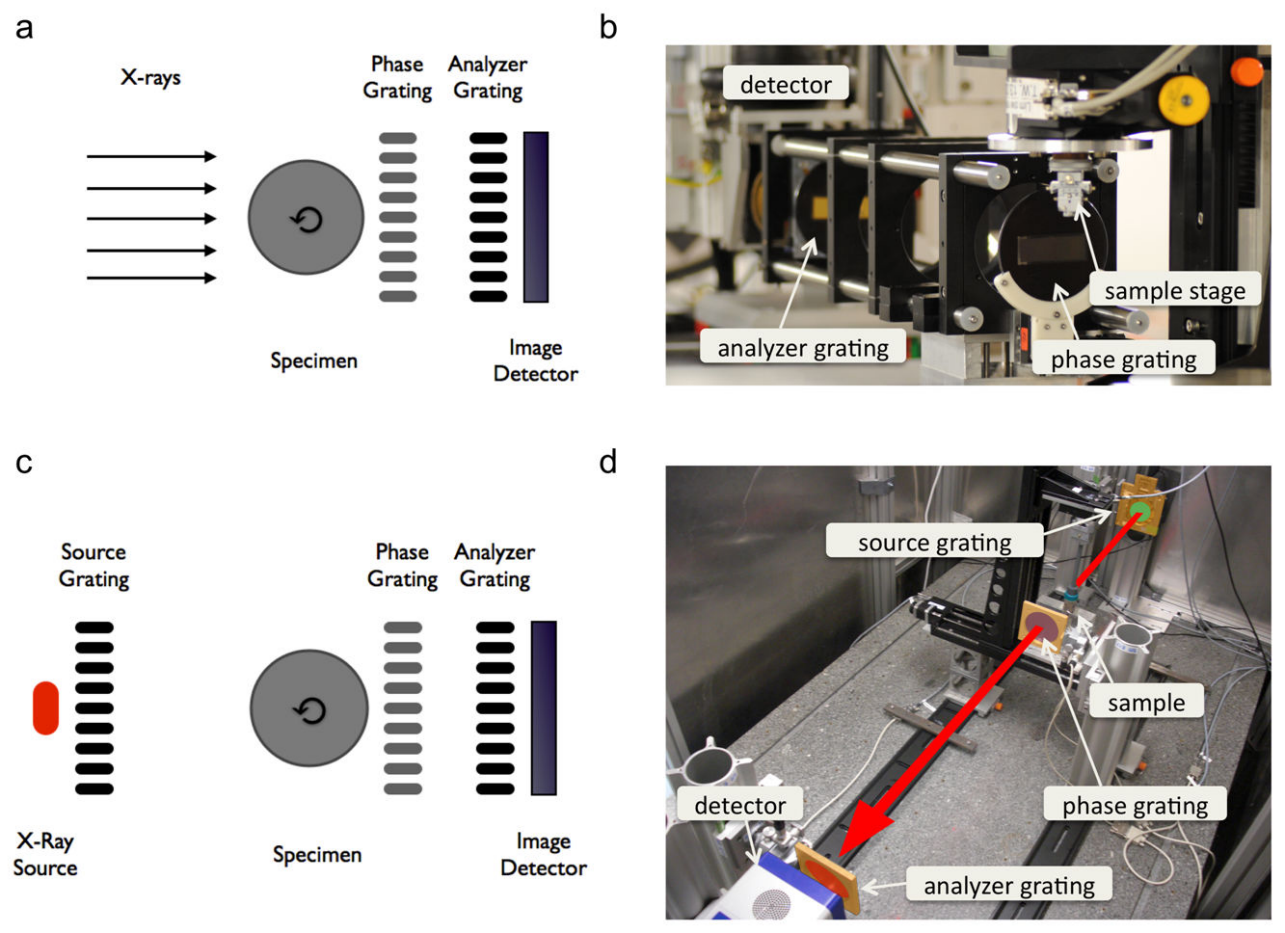
Schematic overview of the experimental setup. (A+B) Schematic drawing and photograph of the talbot interferometer set-up at the synchrotron beamline ID19, ESRF, France. Monochromatic, coherent X-rays travel through the sample and phase distortions are evaluated by a phase grating and an analyzer grating located in between sample and detector. (C+D) Schematic drawing and photograph of the talbot-Lau interferometer set-up used in the laboratory. X-rays originating from a conventional X-ray tube are primed by passing through an additional source grating. The remaining se-tup is analogous to Figure 1A+1B. The red arrow indicates the beam direction.

The principle of grating-based phase-contrast computed tomography (PC-CT) is illustrated in Figure 1a+b and explained in detail by Weitkamp et al. [7]. Briefly, the experimental set-up consists of an X-ray source, a grating interferometer, the sample, and a detector. The grating interferometer for a standard X-ray tube source consists of three gratings: a source grating, a phase grating, and an analyzer grating. The source grating represents an array of slits, which increases beam coherence (=the ability to interfere). Due to the Talbot effect, the phase grating produces interference patterns in discrete distances downstream the X-ray beam. These patterns are usually too small to be directly resolved by a standard X-ray detector with pixel sizes in the range of 50-100 µm, but are detected here by using, a phase-stepping method. For every projection at least three images at different positions of the analyzer grating perpendicular to the beam direction are recorded. From the intensity variations in each pixel during this grating scan, three different signals are calculated from the same set of images: the conventional absorption-contrast, the phase-contrast, and the dark-field signal. While the phase and the absorption images were used for tomographic reconstructions, dark-field information was not explored further in this study. The experimental set-up at the brilliant synchrotron radiation source was similar, but simplified to a two-grating interferometer, as the source grating is not needed.

### Synchrotron set-up

Two carotid artery specimens were inserted in plastic tubes, covered with 10% neutral buffered formalin and measured at the Beamline ID19 of the ESRF, Grenoble, France. The set-up was composed of an X-Ray source, the specimen, a grating-interferometer of the Talbot type (phase grating and an analyzer grating) and the detector, as described above. The monochromatic, coherent X-ray beam with 23 keV was produced by a wiggler and a double-crystal monochromator. Tubes were immersed in a water bath and positioned in front of the grating interferometer. The grating-interferometer was located 145 m from the source and consisted of a silicon phase grating (4.8 µm grating period; 29,5 µm height; Laboratory for Micro- and Nanotechnology, Paul Scherrer Institute Villigen, Switzerland) positioned behind the specimen and a gold analyzer grating (2.4 µm grating period; 74 µm height; Institute for Microstructure Technology and Karlsruhe Nano Micro Facility, Karlsruhe Institute of Technology, Eggenstein-Leopoldshafen, Germany) about 3 cm in front of the detector-system (see Figure 1a+b). Distance between the gratings was 48.1 cm. The detector was a lens-coupled 125 µm LuAG scintillator and a fast-readout, low noise charge-coupled device (CCD) system with an effective pixel size of 5.4 x 5.4 µm^2^ (2048 x 2048 pixels with a physical pixel size of 14 µm). Specimens were rotated by 360° and a total number of 999 projections were obtained for a full tomographic scan. Estimated radiation dose per projection was 10-40 mGy. Once in every 100 projections, 10 reference images without the sample were acquired for flat field correction. A set of four images was recorded for every projection at different positions of the phase grating perpendicular to the beam. Absorption and phase contrast signal were acquired simultaneously and calculated from the intensity variations in each pixel during the grating scan. A standard filtered back projection with a Ram-Lak filter was used to reconstruct the absorption contrast and an imaginary Hilbert filter was used to reconstruct the phase contrast data [12]. Details of the PC-CT image acquisition parameters for each specimen can be found in table 1.

**Table 1:** Acquisition parameters of Phase Contrast CT.

Sample number	Set-Up	Energy (keV) / Tube Voltage (KVp)	Projections / steps / exposure time (sec)	Covered height (mm)	N	Scan time (h)	Water bath	Pixel size (µm)
1	Synchrotron	23 / n.a.	999/4/1	6.5	300	4	Yes	5.4
2	Synchrotron	23 / n.a.	999/4/1	6.5	300	4	Yes	5.4
3	Lab	n.a. / 35	1200/11/5	195	195	24	No	100
4	Lab	n.a. / 35	1200/11/5	535	535	72	No	100
5	Lab	n.a. / 40	1200/11/5	195	195	24	Yes	100

N = Number of slides; n.a. = not applicable

### Set-up at a conventional X-ray tube

Three carotid artery specimens were inserted in plastic tubes, covered with phosphate-buffered saline (PBS) and measured at a laboratory-based set-up. In short, the set-up consisted of a conventional X-ray tube, specimen, grating-interferometer of the Talbot-Lau type (source grating, phase grating and an analyzer grating) and detector (see Figure 1c+d). The X-ray source was an ENRAF Nonius rotating molybdenum anode X-ray tube with a tube voltage of 35 kVp (2 specimens) or 40 kVp (1 specimen) and a tube current of 70 mA. For data acquisition, tubes were either immersed in a water bath (one sample) or measured in air. All three gratings were manufactured by Microworks (Karlsruhe, Germany) with periods of 5.4 µm. The heights of both gold gratings, the source and the analyzer gratings, were roughly 50 µm, the nickel phase grating had a height of 8 µm. Inter-grating distances in this symmetric set-up were 87.5 cm. The interferometer was installed 60 cm from the X-ray source. To allow PC-CT with the conventional non-coherent, non-monochromatic X-ray tube the source gold grating was placed close to the X-ray tube [11,12]. The carotid artery specimen was positioned between source grating and phase grating. It was mounted 7 cm in front of the phase grating, which induced a phase-shift of π to incoming X-rays assuming a mean energy of 22.8 keV. The analyzer grating was positioned behind the phase grating and 4 cm in front of the detector. A single photon counting detector (Pilatus II, Dectris, Baden, Switzerland; 487x195 pixels, 172x172 μm^²^ pixel size) was used. With respect to all parameters of the set-up a sample magnification of 1.72 and an effective pixel size of 100x100 μm^²^ were reached. 1200 projections over 360° were recorded for a tomographic scan with specimen and every 20 projections 5 reference projections without specimen were recorded. Estimated radiation dose per projection was 5-15 mGy. The source grating was stepped over one period for each projection and reference projection while 11 images were acquired. A standard filtered back projection with a Ram-Lak filter was used to reconstruct the absorption contrast and an imaginary Hilbert filter was used to reconstruct the phase contrast data [12]. Details to PC-CT image acquisition parameters and acquisition time for each specimen can be found in table 1.

### Absorption CT acquisition

As mentioned above, the conventional absorption-contrast and the phase-contrast signal are both calculated from the intensity variation in each pixel during the grating scan (phase stepping). This means that absorption and phase-contrast CT images were acquired simultaneously, with the same image acquisition parameters and acquisition times for each specimen (see table 1).

### Image Analysis PC-CT and CT

Data from both set-ups were stored digitally in DICOM format. Slice thickness was 5.4 µm for the synchrotron and 100 µm for the laboratory-based set-up. Absorption and phase contrast images were analyzed using OsiriX 4.0 (32 bit). Vessel lumen, tunica intima, tunica media and tunica adventitia were identified by two experienced radiologists (T.S., H.H.). Window settings were adapted flexibly for optimum evaluation of the different vessel wall layers.

To assess the ability of PC-CT to discriminate tunica intima and tunica media, a 4-point scoring system was applied. A score of 1 indicated that differentiation of tunica intima and media was not feasible; scores of 2, 3 and 4 indicated a moderate, good and excellent differentiation of tunica intima and tunica media, respectively. Every slice of the synchrotron- and lab-based data was scored by two independent reviewers blinded to histology. In case of disagreement, a consensus was reached between both reviewers. Only slices with a score of ≥ 2 were used for quantitative analysis. Lumen-area, wall area, total vessel area, intima and media areas were manually traced in the DICOM images. To evaluate the inter-reader reproducibility of PC-CT area measurements, quantitative measurements were performed by two independent reviewers. To determine contrast quality, the signal-to-noise-ratio (SNR) between vessel wall layers (intima and media) and background material was derived in both, the phase-contrast and the absorption-contrast data by identifying regions of interest (ROIs) in the intima, the media and the surrounding fluid. Mean values and standard deviations from these ROIs as obtained from OsiriX were used to calculate SNRs [17].

### Histology Processing

Carotid specimens were decalcified, and embedded en bloc in paraffin. Serial sections (10 µm thick) were taken every 2 mm throughout the length of the specimen, and stained with haematoxylin-eosin and Movat’s Pentachrome.

### Image analysis histology

Images were digitized by Mirax Scan 150 (Carl Zeiss, Jena, Germany) using a dedicated camera (AVT Stingray F-146C Fire Wire CCD; Pixel resolution 0.23 μm). Image analysis was performed on MIRAX Viewer, Version 1.12.22.1 (Carl Zeiss, Jena, Germany). Vessel lumen, tunica intima and tunica media were identified by an experienced pathologist (U.S.) who was blinded to the PC-CT images. The same parameters as described in the “Image analysis PCI” section were calculated for histology.

### PCI and Histology Matching

Anatomical landmarks such as the relative distance from the common carotid bifurcation and gross morphological features such as lumen size and shape, wall size and shape, plaque configuration, as well as calcifications were used for matching corresponding PC-CT and histology sections [18].

### Statistical Data Analysis

Continuous variables are presented as mean ± standard deviation and binary variables are given in percentages. We performed comparisons between measurement of lumen, intima and vessel wall area for both the synchrotron and the laboratory-based sources and histopathology by taking into account clustering effects among slices per coronary vessels. Vessel dimensions of each modality were compared by fitting mixed effects linear models in SAS Proc Mixed (SAS Institute, Cary, North Carolina) by assuming a standard variance components pattern of covariance. Similarly, we determined whether the absolute and relative degree of overestimation was dependent (predictive) of the size of the measured vessel dimension. To determine inter-reader reproducibility, the intra-class correlation coefficient (ICC) was derived based on measured areas from all individual histology and PC-CT images. As the data was only slightly skewed and by applying the central limit theorem paired student’s t-test was used to assess differences of the SNR between PC-CT and absorption CT images.

All statistical analysis was performed using SAS (SAS Institute, Cary, North Carolina) and a p-value of <0.05 was considered to indicate statistical significance.

## Results

Overall, five carotid artery autopsy specimens, including common, internal and external carotid arteries, were harvested, underwent grating-based CT, and were histologically processed, resulting in a total of 55 cross-sections available for analysis.

### Qualitative Assessment of Vessel Layers and Plaque Characteristics

**Figure 2:**
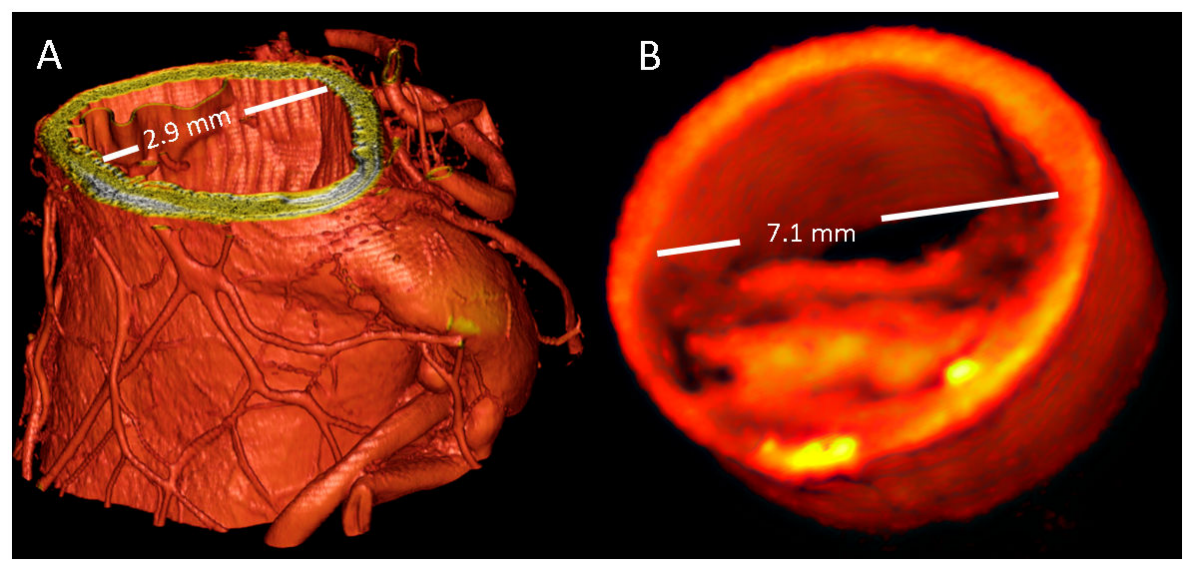
Three-dimensional renderings of the phase contrast tomographic data sets. (A) Near-normal carotid artery imaged at the synchrotron beamline ID19, ESRF, France. Due to the high resolution of the images and the excellent soft tissue contrast, several vasa vasorum can be identified without the use of contrast dye. (B) Atherosclerotic lesion imaged at the laboratory (Images were rendered using VGStudioMax 2.1, Volume Graphics, Heidelberg, Germany)..

**Figure 3:**
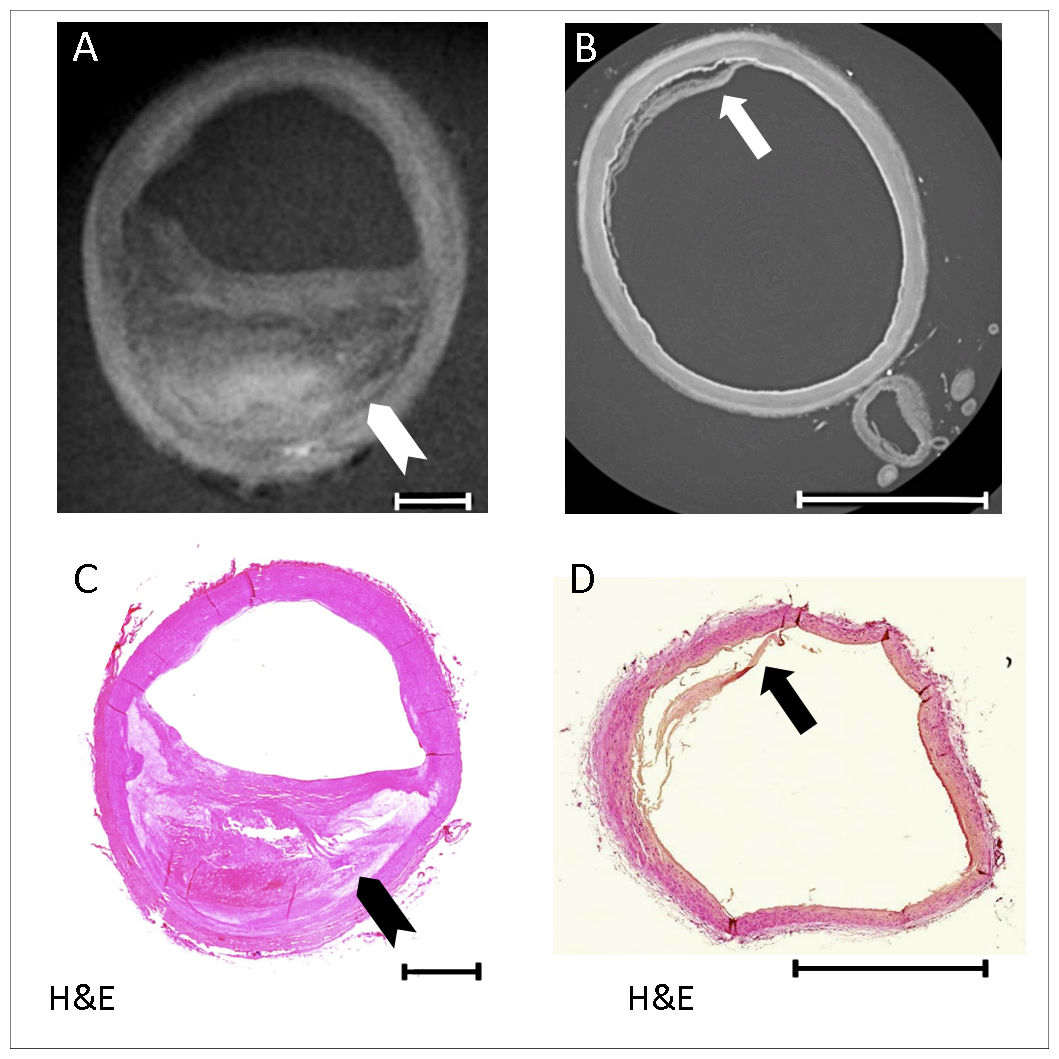
Comparison of PC-CT images recorded at synchrotron and laboratory sources. (A) Axial reconstructed PCI slice from the conventional X-ray tube of the common carotid artery with a large atherosclerotic plaque. The chevron points to a large lipid-rich necrotic core covered by a fibrous cap;. (B) Axial reconstructed PCI slice from the synchrotron facility of the distal external carotid artery with moderate intimal thickening; the arrow points at a detachment of the intimal layer. (C) and (D) corresponding histology sections (PCI = Phase Contrast Imaging; Length of the scale bar = 2 mm).

Three-dimensional threshold-rendered quantitative PC-CT images of an external carotid artery from a specimen imaged at the synchrotron set-up and of a part of the common carotid artery from a specimen imaged at the laboratory set-up are shown in Figure 2a and 2b, respectively. Visual inspection revealed that images obtained at the synchrotron radiation facility provided high contrast and resolution, allowing for sufficient differentiation of the individual vessel layers and visualization of very small anatomic details such as the vasa vasorum without the use of any contrast dye (Figure 2a). In contrast, images obtained at the laboratory-based set-up resulted in images of lower resolution, but provided sufficient image contrast to reveal detailed information on the different vessel layers and the different plaque components, such as a fibrous cap covering a large lipid / necrotic core (Figure 2b). Representative cross-sectional images with corresponding histopathology sections for both imaging set-ups are provided in Figure 3, illustrating the ability to differentiate individual vessel wall layers by both PC-CT acquisitions.

The ability to differentiate vessel wall layers was higher for the synchrotron set-up as compared to the laboratory-based set-up (average score 4.0 ± 0 vs. 2.9 ± 0.9, p<0.05). Specifically, all slices (10/10) of the images obtained at the synchrotron were classified with the highest ability to differentiate between layers (score of 4). None of the images obtained at the synchrotron had to be excluded. At the laboratory-based set-up 13 out of 44 slices (29.5%) received the highest score of 4, 18 out of 44 sections (40.9%) received a score of 3 and 10 out of 44 sections (22.7%) received a score of 2. In 3 out of 44 slices (6.8%) obtained at the laboratory set-up, image quality was insufficient to differentiate between intima and media. These were excluded from further quantitative analysis. Low scores of ≤2 were found near the bifurcation (n=5), near areas of extensive atherosclerosis with extensive calcification (n=7), or in parts with artificial damage to the vessel wall (n=1).

### Quantitative Measurements of Vessel Layers

All areas measured by PC-CT were significantly greater for both synchrotron- and laboratory-based images as compared to histopathology (all p<0.01, range 10-30%, Table 2), except the tunica media measured on a synchrotron source (1.8±0.6 vs. 1.8±0.6, p=0.8), which was not different.

**Table 2:** Quantitative Vessel Wall Measurements.

**Variable (Areas)**	**Histology [mm^2^]**	**PC-CT [mm^2^]**	**P-Value**
***Synchrotron(n=10)***			
Lumen	4.6 ± 3.1	6.0 ± 3.2	<0.001
Wall	2.1 ± 0.8	2.3 ± 0.8	<0.001
*Tunica* *intima*	*0.3 ± 0.2*	*0.5 ± 0.2*	*<0.001*
*Tunica* *media*	*1.8 ± 0.6*	*1.8 ± 0.6*	*0.8*
Total vessel area	6.7 ± 3.9	8.2 ± 4.0	0.03
***ConventionalX-raytube(n=41)***			
Lumen	18.9 ± 13.9	25.0 ± 17.5	<0.001
Wall	23.5 ± 16.0	34.9 ± 23.0	<0.001
*Tunica* *intima*	*16.3 ± 14.4*	*22.2 ± 18.8*	*<0.001*
*Tunica* *media*	*7.4 ± 3.9*	*12.6 ± 5.1*	*<0.001*
Total vessel area	40.0 ± 27.8	55.7 ± 36.5	<0.001

Comparison of areas (in mm^2^) of lumen, wall, tunica intima, tunica media and total vessel as determined using histology or PC-CT images recorded at either synchrotron or laboratory source. All values given as mean ± standard deviation.

(grating-based PC-CT = phase-contrast computed tomography)

For the synchrotron set-up, the mean wall area was 2.3±0.8 mm^2^ in PC-CT and 2.1±0.8 mm^2^ in histology. Despite the differences in absolute numbers, there was a strong correlation between PC-CT and histology measurements and thus all measurements of vessel areas on synchrotron PC-CT were significant predictors of vessel dimensions on histology. While the measurement of intima area on PC-.CT was the weakest predictor (slope: 0.91 per mm^2^, 95%-CI: 0.64-1.17), all other measurements had a slope >0.96 (Table 3) and were strongly predictive for histology measurements. The predicted difference between the measurements of the tunica intima was 0.14±0.87 mm^2^ and significant (95%-CI: 0.09 to 0.19). The absolute overestimation of the tunica intima by PC-CT increased with increasing area on histology, but this association was not significant (slope: 0.19, 95%-CI: -2.1 to 2.5).

**Table 3:** Prediction of vessel areas as determined by histopathology by PC-CT.

	**Synchrotron PC-CT**		**Laboratory PC-CT**	
	**Slope**	**95%-CI**	**Slope**	**95%- CI**
N	10		41	
Lumen Area	0.96	0.90-1.02	0.76	0.69-0.83
Wall Area	0.99	0.80-1.17	0.68	0.63-0.73
*Tunica* *Media*	*1.00*	*0.82-1.18*	*0.53*	*0.35-0.70*
*Tunica Intima*	*0.91*	*0.64–1.17*	*0.73*	*0.66-0.80*
Total Vessel Area	0.97	0.89-1.06	0.74	0.70-0.79

Predicted histology vessel areas by synchrotron-based PCI and laboratory-based PCI with pertaining 95%-confidence intervals (95%-CI).

(grating-based PC-CT = phase-contrast computed tomography)

For the laboratory grating-based set-up, the mean wall area was 32.6±20 mm^2^ in PC-CT and 22.6±16.2 mm^2^ in histology. All measurements of vessel areas on laboratory-PCI were significant predictors of vessel dimensions on histology but generally weaker than the synchrotron-based measurements (Table 2). While for the laboratory-based PC-CT, measurement of the lumen area was the strongest predictor of the histology measurement (slope: 0.76 per mm^2^, 95%-CI: 0.69 to 0.83), measurement of the tunica media was the weakest predictor of the histology measurement (slope: 0.53 per mm^2^, 95%-CI: 0.35 to 0.70, Table 3). The predicted difference between the measurement of the tunica media was 5.17±3.7 and significant (95%-CI: 4.03 to 6.3). Also, the absolute overestimation of the tunica intima by PC-CT increased weakly with increasing area on histology, but this association was not significant (slope: 0.10, 95%-CI: -0.24 to 0.43).

### Signal Characteristics Quantitative Measurements of Vessel Layers

The SNR of intima and media were significantly higher on synchrotron PC-CT images as compared to corresponding absorption CT images (SNR: 4.9 vs. 0.2; p<0.001 and 10.0 vs. 0.03 p<0.001, respectively). Similarly, the SNR of intima and media were significantly higher on PC-CT images obtained with the conventional X-Ray tube as compared to corresponding absorption CT images (4.1 vs. 1.0; p<0.001 and 6.1 vs. 0.9; P<0.001, respectively).

### Reproducibility

Inter-reader reproducibility was excellent for synchrotron-based area measurements of lumen, wall, total vessel area, intima and media (all ICCs >0.98, see Table 4). Similarly, inter-reader reproducibility was strong for lab-based area measurements of lumen, wall, total vessel area, intima and media (all ICCs >0.87, see Table 4).

**Table 4:** Inter-reader reproducibility of PC-CT measurements recorded at either synchrotron or laboratory source.

	**Synchrotron PC-CT**		**Laboratory PC-CT**	
	**ICC**	**95% CI**	**ICC**	**95% CI**
N	10		41	
Lumen Area	0.99	(0.98-1.00)	0.99	(0.99-1.00)
Wall Area	0.98	(0.99-1.00)	0.98	(0.96-0.99)
Tunica Media	0.99	(0.98-1.00)	0.87	(0.77-0.92)
Tunica Intima	0.98	(0.99-1.00)	0.97	(0.94-0.98)
Total Vessel Area	0.99	(0.98-1.00)	0.99	(0.98-1.00)

(grating-based PC-CT = phase-contrast computed tomography; ICC = Intra Class Correlation Coefficient; CI = Confidence Interval)

## Discussion

**Figure 4:**
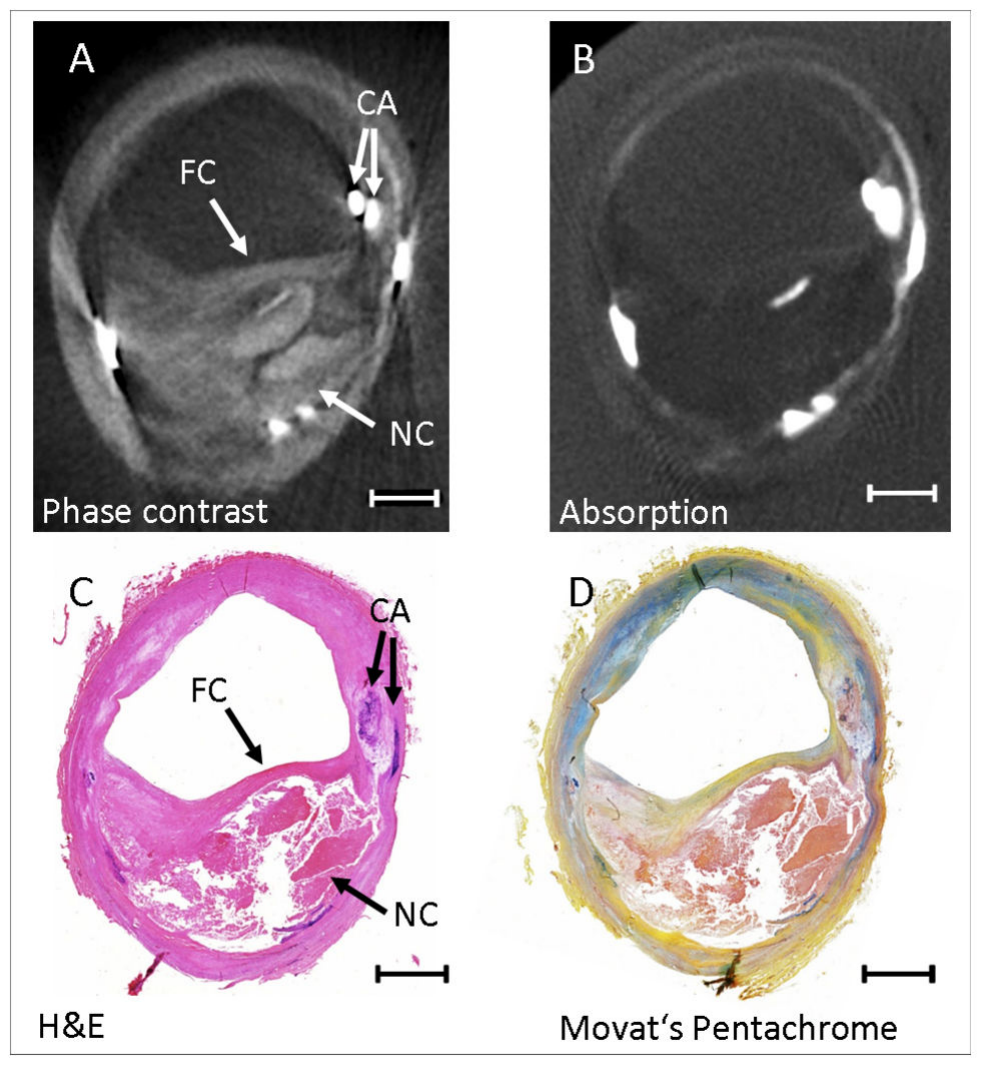
Comparison of PC-CT images and absorption CT images obtained with a conventional X-ray tube; and corresponding histology sections. (A) Axial reconstructed PC-CT slice from the conventional X-ray tube, (B) axial reconstructed CT slice from the conventional X-ray tube, (C) and (D) corresponding histology sections. This atherosclerotic lesion shows a large lipid-rich necrotic core and a relatively thin fibrous cap. The arrows point to the fibrous cap (FC), the lipid-rich necrotic core (NC) and an area of calcification (CA). Several other calcifications are seen in this specimen (PC-CT = Phase Contrast Computed Tomography; CT = Computed Tomography, Length of the scale bar = 2 mm).

In this proof-of concept study, our results indicate that grating-based PC-CT of carotid atherosclerotic plaque is feasible using both, synchrotron radiation and conventional laboratory-based X-ray sources. Both experimental set-ups generated images that allowed for quantification of vessel dimensions, including lumen, wall and total vessel wall areas in strong correlation but significant overestimation in comparison with histopathology. Also, our results suggest that PC-CT enables differentiation between tunica intima and tunica media, indicating improved soft tissue contrast compared to all other clinically available vascular imaging techniques, which usually fail to discriminate between these two vessel wall layers. In addition, signal-to-noise ratios were higher for both PC-CT techniques compared to conventional absorption CT, which indicates its superior contrast compared to absorption CT and its potential ability for atherosclerotic plaque characterization, i.e. for the detection of different components of high-risk atherosclerotic lesions (Figures 2b & 4). Furthermore, we were able to visualize the vasa vasorum in detail without the use of contrast dye (Figure 2a), which could be an interesting application for luminal stenosis imaging.

In our study, we specifically report on the translation of the grating-based phase-contrast technique from a synchrotron radiation source to a laboratory-based set-up. While synchrotron sources enable acquisition of PC-CT images with high resolution due to a monochromatic X-ray beam with high photon flux, conventional X-ray sources provide a polychromatic X-ray beam with less photon flux. In order to achieve sufficient image quality with the conventional X-ray tube despite the loss in photon flux we used a lower spatial resolution. Our findings indicate that despite the lower spatial resolution at the conventional X-ray tube (100 vs. 5.4 µm) excellent soft-tissue contrast of atherosclerotic vessels can be maintained. This may represent a critical step in the development of the technology, as it enables a pre-clinical research setting independent of large synchrotron facilities, with the potential of standardized repeat acquisitions of different models and experimental in-vivo imaging. While not yet applicable to patients, the current value of the technique lies in non-invasive characterization of atherosclerotic disease to further understand the pathophysiological disease process and is a basis for further technical developments. More importantly, in this pre-clinical laboratory setting, critical aspects of the technique, such as optimized acquisition parameters, reconstruction algorithms, and dose reduction approaches can be pursued. These aspects will be essential to establish atherosclerotic phase-contrast imaging in humans and will require further research efforts.

Given its early stage, there are only few studies reporting on the application of phase-contrast imaging to atherosclerotic vessels to date. Shinohara et al. [19] investigated plaque components of Apo lipoprotein E-deficient mice using a crystal interferometer at a synchrotron radiation source. Takeda et al used a monolithic X-ray interferometer for phase-contrast x-ray imaging of atherosclerotic lesion volumes and plaque components of the brachiocephalic artery of Apo lipoprotein E-deficient mice with or without anti-inflammatory therapy [20]. Appel et al. [21,22] used analyzer-based phase-contrast x-ray imaging to image the microstructure of carotid plaques. Taken together, their results suggest a great potential of X-ray phase contrast imaging in visualization of plaque components and detection of unstable aortic and carotid plaques with a spatial resolution of 10-20 μm. However, all of these studies relied on a brilliant synchrotron radiation source, with the restrictions mentioned above. Thus, these phase-contrast imaging methods cannot easily be transferred to clinical imaging.

We confirm these initial observations on the ability of the technique for plaque characterization, but extend their findings substantially. Our results similarly indicate that plaque features, such as the fibrous cap and the lipid-rich necrotic core, can be detected and that PC-CT furthermore permits quantification of lumen, wall and total vessel wall areas with excellent correlation to histopathology. Notably, we were also able to demonstrate that synchrotron-based PC-CT is able to reliably differentiate between tunica intima and tunica media which cannot be discriminated by CT, MRI or ultrasound. The same was true for the lab-based measurements although differentiation between vessel layers was more difficult. This might in part be attributed to the lower resolution. However, the vessels which were examined in the laboratory set-up showed a very advanced stage of atherosclerosis with large calcifications, lipid-rich necrotic core and signs of inflammation, making it more difficult to discern tunica, intima and media, even in histology. In contrast, the vessels analyzed in the synchrotron set-up showed only minor disease.

While our PCI provides images beyond the resolution of CT or MRI, there are other high-resolution - but invasive - catheter-based imaging modalities such as optical coherence tomography (OCT) or infrared-spectroscopy, which have shown to be of value for the detection of high-risk plaque features including fibrous cap thickness and are currently employed in clinical trials [23,24].

While we observed a strong correlation of measurement of plaque dimensions by PCT with histology, our results also indicate that there was significant overestimation of the obtained measurements (predicted relative differences for both synchrotron and laboratory-based set-up) when compared with histopathology. This finding is in line with prior research on novel imaging modalities for vessel wall quantification (i.e. high-resolution MRI of carotid arteries or CT or the coronary arteries) and has been attributed to plaque shrinkage of 19% to 35% due to histological processing and limited spatial resolution to a smaller amount [18,25].

The energy of 23 keV used at the synchrotron radiation source and the tube voltage of 35 kVp at the lab-based set-up was chosen because gratings were optimized for such a low energy range. In contrast, conventional CT-scans are usually performed at a tube voltage of about 100-120 kVp [25], resulting in mean photon energies between 50 and 80 keV. To increase the design X-ray energy, grating structures with very high aspect ratios are required, which have been presented in a feasibility study by Grund et al [26]. Further technical advances are necessary for optimal image acquisition at higher photon energies. Although initial results are promising [27] it has to be assessed further whether high photon energies provide images with sufficient contrast for visualization of vessel components.

It is important to note that the absorption CT, which we obtain with our method, is not directly comparable with clinically available full-body absorption CT scans which are optimized to scan large objects with a relatively low radiation dose and a short acquisition time. As we were studying small ex-vivo specimens, the clinically available full-body CT would not lead to sufficient results neither in terms of contrast nor in spatial resolution. However, the X-ray energies we used for this study were similar – although not identical - to the optimum X-ray energy for small specimens when working with monochromatic X-ray sources, as proposed by Grodzins et al. [28].

Importantly, there are a number of aspects that were not addressed in this first feasibility study but will need to be addressed in the future. The role of contrast agents in phase-contrast imaging remains unclear. The observed differences in soft-tissue contrast were obtained without the use of any contrast agent. Besides iodinated contrast agents there are a number of other substances that portrait a high index for photon phase-shift to occur and may further enhance soft-tissue resolution. For example high contrast for vessel lumenography by phase-contrast imaging can be achieved using normal saline in liver vessels [29]. Future, systematic research will be necessary to address this issue.

### Limitations

Rigorous quantitative comparison of PC-CT images and histology sections requires careful attention to registration of cross-sections. Because the lab-based PC-CT slice thickness (100 µm) is greater than that of histological sections (10 µm), one PC-CT slice represents a composite of 10 histology sections. Thus, in complex specimens containing lesions that may change significantly in size or composition from section to section, it can be difficult to obtain precise co-registration. Also, the size of the vessels imaged at each set-up was significantly different (smaller at the synchrotron-based set-up). While this approach is attributable to the limited field-of-view and the initial nature of our results, upcoming, more systematic research will need to address this potential more thoroughly by employing identical vessel specimens for both imaging set-ups.

Absorption CT data was obtained with a scan protocol, which is not comparable with any clinical atherosclerosis imaging protocol. Therefore, the presented SNRs of the absorption CT are likely to differ compared to SNRs obtained with a clinically used CT imaging protocol. However, the grating-based absorption CT and the PC-CT images were obtained simultaneously under exactly the same conditions and it is very likely that SNRs of PC-CT could be further increased with increasing experience with this relatively new technique. Moreover, PC-CT was able to differentiate between the tunica intima and the tunica media and this differentiation is not possible with preclinical or clinical absorption CT, thus underlining the superior soft tissue contrast of PC-CT compared to existing techniques.

The estimated radiation doses used in this study were much higher than radiation doses used for clinical absorption CT imaging. We are confident, however, that a significant reduction in applied radiation dose can be achieved with our conventional X-ray tube setup by applying the following: i) optimization of the setup in terms of adequate spectrum filtering; ii) application of higher photon energies; iii) reduction of the number of phase encoding steps or application of new acquisition schemes, e.g. advanced stepping methods [30,31]; iv) reduction of acquisition time; v.) increase in pixel size and vi) use of detectors with a higher detection efficiency. Furthermore, new iterative reconstruction methods for phase contrast imaging are currently under development which will further decrease the dose. However, in this study we aimed to evaluate a potential application of phase contrast CT and to demonstrate that this technique is able to provide additional information compared to existing imaging techniques. This ex vivo feasibility study of human arteries was a first step in this direction.

It should be noted that our results only apply to ex-vivo, formalin fixated specimens. Due to security regulations at the synchrotron radiation facility, we were unable to measure unfixed samples. Currently there is no evidence about the influence of formalin fixation on the signal characteristics of arterial vessel layers in phase-contrast imaging; however, this aspect will be investigated in upcoming experiments at the laboratory-based set-up.

With regard to a potential future in-vivo application, e.g. scanning of the human neck, it is important to mention that image quality may be affected by X-ray scattering near bones. The resulting artifacts are comparable to those of metallic implants in clinical attenuation-based CT and might result in a loss of information downstream the beam. Current approaches to reduce these artifacts include iterative reconstruction methods (e.g. maximum likelihood methods [32]), which allow for non-equidistant sampling (e.g. skipping angular projections in which scattering was too high), and reduction of the sensitivity of the imaging system to X-ray scattering by increasing the X-ray energy [27].

Finally, it remains to be determined if similar image quality can be achieved in a potential human in-vivo setting at acceptable radiation doses.

## Conclusions

The results of this study indicate that detailed atherosclerotic plaque imaging with enhanced soft-tissue resolution is feasible using both synchrotron and laboratory grating-based PC-CT. Specifically, our results demonstrate that both experimental set-ups generate images that allow for differentiation of the tunica intima and tunica media and potentially the detection of high-risk atherosclerotic plaque components, such as the fibrous cap and the lipid / necrotic core. Thus, our results provide evidence that grating-based phase-contrast imaging with conventional X-ray tubes is feasible and has high potential for atherosclerotic plaque characterization. Further studies are needed to test whether this technique can be used to identify and quantify the key components of the vulnerable atherosclerotic plaque.
